# tRNA^Glu^ Increases the Affinity of Glutamyl-tRNA Synthetase for Its Inhibitor Glutamyl-Sulfamoyl-Adenosine, an Analogue of the Aminoacylation Reaction Intermediate Glutamyl-AMP: Mechanistic and Evolutionary Implications

**DOI:** 10.1371/journal.pone.0121043

**Published:** 2015-04-10

**Authors:** Sébastien P. Blais, Jack A. Kornblatt, Xavier Barbeau, Guillaume Bonnaure, Patrick Lagüe, Robert Chênevert, Jacques Lapointe

**Affiliations:** 1 Département de Biochimie, de Microbiologie et de Bio-informatique, Université Laval, Québec, Canada; 2 Institut de Biologie Intégrative et des Systèmes (IBIS), Université Laval, Québec, Canada; 3 Département de Chimie, Université Laval, Québec, Canada; 4 The Quebec Network for Research on Protein Function, Structure, and Engineering (PROTEO), Québec, Canada; 5 Department of Biology, Centre for Structural and Functional Genomics, Faculty of Arts and Science, Concordia University, Montréal, Canada; Institute of Molecular Genetics IMG-CNR, ITALY

## Abstract

For tRNA-dependent protein biosynthesis, amino acids are first activated by aminoacyl-tRNA synthetases (aaRSs) yielding the reaction intermediates aminoacyl-AMP (aa-AMP). Stable analogues of aa-AMP, such as aminoacyl-sulfamoyl-adenosines, inhibit their cognate aaRSs. Glutamyl-sulfamoyl-adenosine (Glu-AMS) is the best known inhibitor of *Escherichia coli* glutamyl-tRNA synthetase (GluRS). Thermodynamic parameters of the interactions between Glu-AMS and *E*. *coli* GluRS were measured in the presence and in the absence of tRNA by isothermal titration microcalorimetry. A significant entropic contribution for the interactions between Glu-AMS and GluRS in the absence of tRNA or in the presence of the cognate tRNA^Glu^ or of the non-cognate tRNA^Phe^ is indicated by the negative values of –TΔS_b_, and by the negative value of ΔC_p_. On the other hand, the large negative enthalpy is the dominant contribution to ΔG_b_ in the absence of tRNA. The affinity of GluRS for Glu-AMS is not altered in the presence of the non-cognate tRNA^Phe^, but the dissociation constant *K*_d_ is decreased 50-fold in the presence of tRNA^Glu^; this result is consistent with molecular dynamics results indicating the presence of an H-bond between Glu-AMS and the 3’-OH oxygen of the 3’-terminal ribose of tRNA^Glu^ in the Glu-AMS•GluRS•tRNA^Glu^ complex. Glu-AMS being a very close structural analogue of Glu-AMP, its weak binding to free GluRS suggests that the unstable Glu-AMP reaction intermediate binds weakly to GluRS; these results could explain why all the known GluRSs evolved to activate glutamate only in the presence of tRNA^Glu^, the coupling of glutamate activation to its transfer to tRNA preventing unproductive cleavage of ATP.

## Introduction

Aminoacyl-tRNA synthetases (aaRS) play a front-line role in protein biosynthesis; they are responsible for the attachment of specific amino acids to their cognate tRNAs. This two-step reaction begins with the amino acid activation by condensation with an ATP molecule, creating an aminoacyl-adenylate (aa-AMP) which then reacts on the aaRS with the 3’-terminal adenosine of the tRNA’s acceptor stem, giving the aminoacyl-tRNA (aa-tRNA) which participates in protein biosynthesis on the ribosome. The essential function of aaRSs in translation makes them promising targets for inhibitors that could be used as antibiotics, such as pseudomonic acid A [1], an inhibitor of isoleucyl-tRNA synthetase (IleRS) used as a topical antibacterial treatment. Recent reviews [2] on the subject show that there are new developments in this field, including pharmacological patents [3].

**Fig 1:**
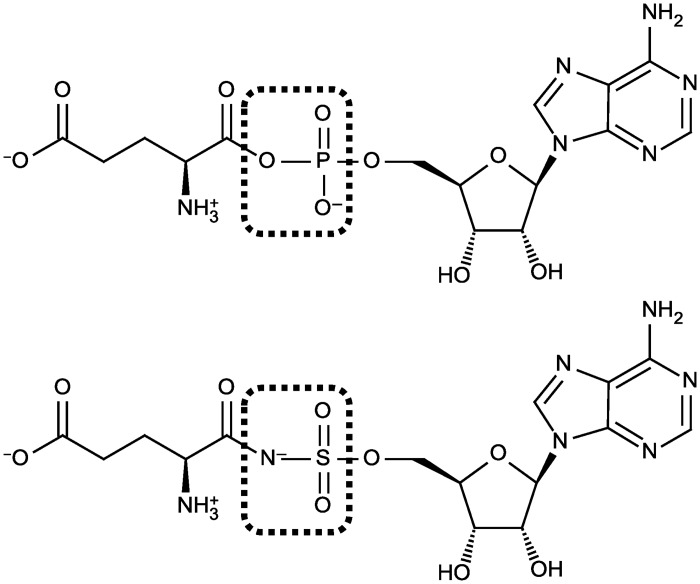
Structures of Glu-AMP and Glu-AMS. Structures of the reaction intermediate Glu-AMP (top), and of its non-hydrolysable analogue Glu-AMS (bottom), which is an inhibitor of *E*. *coli* GluRS [6].

Several types of stable analogues of aa-AMP inhibit aaRSs (reviewed by Chênevert et al., 2003, and by Finn and Tao, 2005) [4,5]. Aminoacyl-sulfamoyl-adenosines are amongst the most potent ones. Glutamyl-sulfamoyl-adenosine (Glu-AMS) (Fig 1) is a competitive inhibitor of *Escherichia coli* glutamyl-tRNA synthetase (GluRS) with a *K*_i_ of 2.8 nM [6] and is 25 times less efficient against murine hepatic GluRS. This result suggests that the structures of the active sites of bacterial and mammalian GluRSs differ significantly, and indicates that Glu-AMS derivatives with bactericidal properties and low toxicity for humans could be developed.

Most aaRS can activate their amino acid substrate in the absence of tRNA; the aa-AMP synthetized by these enzymes are relatively stable, which allows the characterization of their binding to their cognate aaRS (for instance, see Fersht (1977) [7] for isoleucyl-adenylate (Ile-AMP) and valyl-adenylate (Val-AMP)). On the other hand, all known GluRSs, glutaminyl-tRNA synthetases (GlnRSs), arginyl-tRNA synthetases (ArgRSs), and class 1 lysyl-tRNA synthetases (LysRSs) (closely linked to GluRSs) [8] do not activate their amino acid substrate in the absence of tRNA, but still catalyze the aminoacylation reaction via a two-step mechanism involving a very unstable aa-AMP intermediate [8–11] (reviewed by Schimmel and Söll, 1979, and by First et al., 2005) [12,13].

The structures of *Thermus thermophilus* GluRS and of its complexes with several substrates and inhibitors [14] revealed that ATP binding by GluRS is switched to the productive mode by tRNA^Glu^ binding [15], and that in the presence of tRNA^Glu^, GluRS recognizes specifically L-glutamate [16], excluding the non-cognate amino acids L-glutamine and D-glutamate which interact with GluRS in the absence of tRNA [17]. The structure of the *T*. *thermophilus* tRNA^Glu^•GluRS•Glu-AMS complex, which may represent the post-transition state of the glutamate-activation reaction, was determined at a resolution of 2.69 Å (PDB ID 2CV2) [16]. The reason for the tRNA-requirement in the activation reaction catalyzed by GluRSs and the three other above-mentioned aaRSs throughout evolution is not yet known.

We report here the values of thermodynamic parameters of the *E*. *coli* GluRS Glu-AMS interaction in the presence of the cognate tRNA^Glu^ or of a non-cognate tRNA^Phe^, or in the absence of tRNA. These values suggest that all the known GluRSs evolved to activate glutamate only in the presence of tRNA^Glu^ to prevent unproductive cleavage of ATP [18]. Moreover, this thermodynamic characterization of the GluRS Glu-AMS interaction (see equation below) could complement structural data for the design of less polar derivatives of Glu-AMS that could have bactericidal activity.

GluRS + Glu-AMS ↔GluRS•Glu-AMS

## Materials and Methods

### Enzyme and tRNA

Overproduction and purification of *E*. *coli* GluRS were performed as previously described [19] with the following modifications. A C-terminal histidine-tagged GluRS was used instead of the N-terminal tagged one. The overproduction was induced overnight at 30°C with 1 mM IPTG. The GluRS was purified to homogeneity, as revealed by SDS-PAGE analysis (result not shown).

Overproduction and purification of *E*. *coli* tRNA^Glu^-enriched total tRNA was done as described [20]. The aminoacylation plateau indicated that the final product contained 26% tRNA^Glu^. *Saccharomyces cerevisiae* tRNA^Phe^, used as a negative control, was purchased from Sigma-Aldrich (cat No: R4018).

### GluRS inhibitor

Glu-AMS (5’-*O*-[*N*-(L-glutamyl)sulfamoyl]adenosine, Trilink Lot #A1004-060606), a stable analogue of the GluRS reaction intermediate Glu-AMP, and a potent inhibitor of *E*. *coli* GluRS with respect to glutamic acid [6] was purchased from RNA-TEC (Leuven, Belgium). A 10 mM stock solution was prepared in Tris-HCl buffer (50 mM, pH 7.9, 10 mM MgCl_2_).

### Isothermal Titration Microcalorimetry

The GluRS solution with or without tRNA was dialyzed overnight in a D-tube dialyzer (Novagen) against 2 L of dialysis buffer (50 mM HEPES-KOH, pH 7.2, 10 mM MgCl_2_) at 4°C with light stirring. The next morning, the dialyzed solution was recovered and the volume adjusted by adding dialysis buffer to obtain the desired concentration (typically 9 μM GluRS and 11 μM tRNA^Glu^). This solution was kept on ice until use. Glu-AMS was diluted in the dialysis buffer to obtain a final concentration of 90 μM. All buffers and solutions were degased with stirring under vacuum. The microcalorimetry experiments were carried out in a VP-ITC 100 microcalorimeter (MicroCal, GE Healthcare) using deionized water as an internal reference for all assays. VPViewer ITC 2000 and Origin v 5.0 softwares (Microcal Software, Inc) were used for data collection and analysis, respectively.

To measure the thermodynamic parameters of the interaction between Glu-AMS and GluRS, in the presence and in the absence of tRNA, a solution containing 9 μM GluRS with or without a stoichiometric excess of tRNA^Glu^ or *S*. *cerevisiae* tRNA^Phe^ (Table 1) was placed in the sample cell of the microcalorimeter. The following conditions were used for all tests: reference power was set at 12 μcal/s and stirring at 300 rpm, the “high” feedback mode and “No check Temp”, “Fast Equil” and “Auto” ITC equilibration options were selected. The 90 μM Glu-AMS solution was loaded in the injection syringe. After a first injection of 1 μL over 2 s, a series of 39 injections (7.4 μL over 14.8 s) with 240 s between injections was performed. Injections of Glu-AMS in GluRS alone, GluRS•tRNA^Glu^ or GluRS•tRNA^Phe^, were performed at 30°C (303 K), and done in duplicate. Injections of Glu-AMS in GluRS were also performed at 20°C and 37°C (293 and 310 K): 14 injections (20 μL over 40 s) followed the first injection of 1 μL. Each temperature was tested in duplicate, and in triplicate at 37°C.

**Table 1:** Influence of tRNA on GluRS/Glu-AMS binding at 30°C.

	*E*. *coli* GluRS	*E*. *coli* GluRS + *E*. *coli* tRNA^Glu^	*E*. *coli* GluRS + *S*. *cerevisiae* tRNA^Phe^
n^a^	0.9998 ± 0.0094	1.0018 ± 0.0041	0.9902 ± 0.0410
ΔH_b_ (cal/mol)	-5041 ± 63	-7990 ± 62	-5173 ± 279
ΔS_b_ (cal/mol·K)	13.19 ± 0.16	10.52 ± 0.08	7.92 ± 0.46
ΔG_b_ (cal/mol)	-9056 ± 38	-11304 ± 209	-8544 ± 309
-TΔS_b_ (cal/mol)	-3996 ± 50	-3186 ± 25	-2398 ± 140

n = stoichiometry coefficient (number of moles of Glu-AMS bound per mole of GluRS monomer), ΔH_b_ = reaction enthalpy, ΔS_b_ = reaction entropy, ΔG_b_ = reaction energy (calculated with the formula ΔG_b_ = -RT Ln *K*_b_, where R = 1.987 cal/mol·K).

All values and errors in this table were obtained by weighting by inverse variance [32], except for ΔG_b_ values and errors, obtained by simple average and standard error calculations.

Raw data and calculated values for each separated ITC runs are shown in S1 Table.

The following nomenclature was used to describe the interaction at equilibrium between Glu-AMS and either GluRS or a GluRS•tRNA complex:

GluRS•Glu­AMS↔GluRS+GluS↔Gl

$$K_{\text{d}}=\left[\text{GluRS}\right]\times \left[\text{Glu-AMS}\right]/\left[\text{GluRS}•\text{Glu-AMS}\right]$$

$$1/K_{\text{d}}=K_{\text{b}}\;\left(binding\;constant,\;sometimes\;referred\;to\;as\;K_{a}\right).$$

### Homology modeling

The primary sequence of *E*. *coli* GluRS (471 residues) was obtained from the UniProt Consortium (2012) (UniProt P04805). Two structures were identified as templates for homology modeling from a standard protein Blast (BLASTP) query using the Protein Data Bank (PDB) database on the NCBI/Blast web server [21]. The two GluRSs identified are from *Burkholderia thailandensis* [22] and *Thermosynechococcus elongatus* [23], with Uniprot Q2SX36 and Q8DLI5, respectively. A multiple sequence alignment of the sequences using the default parameters of T-Coffee v10.00.r1613 Build 432 [24] showed similarities of 64.7% and 54.3% between the *E*. *coli* GluRS and the *B*. *thailandensis* and *T*. *elongatus* GluRSs, and identities of 49.7% and 41.3%, respectively (the multiple alignment is given in S1 Fig). However, several sections of the *B*. *thailandensis* GluRS crystal structure are missing. Consequently, the *T*. *elongatus* GluRS structure (PDB 2CFO) was chosen as the template for homology modeling.

The models were built using the T-Coffee alignment and MOE [25] with default parameters, as described previously [26], with the exception that 10 different side chain positions were explored for each model. The best models were validated using MolProbity [27], and the model with the highest score was used for system preparation and docking simulations. Then, three important water molecules (residues 2001, 2007 and 2009 of 2CFO) were added to the model according to 2CFO and 2CV2 crystal structures. In addition, new conformations of Met-250 were generated using the rotamer explorer tool in MOE, as this residue was pointing toward the solvent and would clash with the tRNA. The lowest energy conformation pointing toward the active site was chosen. Then, the Glu-AMS from 2CV2 was added to the modeled structure. This receptor was prepared with the MOE LigX tool to adjust the hydrogen atoms, the rotamers and to minimize the system's energy as previously described [28]. Two models were then built, with and without the tRNA (hereafter referred to as *E*. *coli* GluRS and *E*. *coli* GluRS•tRNA^Glu^, respectively). The *E*. *coli* GluRS•tRNA^Glu^ model was built by adding the tRNA^Glu^ from PDB 2CV2 to the *E*. *coli* GluRS model. The tRNA^Glu^ conformation was energy minimized while keeping fixed all the other atoms. For both models, Open Babel 2.3.2 [29,30] was used to convert pdb files in pdbqt and assign Gasteiger charges.

### Docking simulations

Glu-AMS has been docked to both *E*. *coli* GluRS and *E*. *coli* GluRS•tRNA^Glu^ models. RMSDs between the best ranked conformation of each system and PDB 2CV2 were calculated for the Glu-AMS heavy atoms. All docking calculations were carried out with Autodock VINA 1.1.2 [31] using a rigid receptor for the protein and some flexibility between nucleotides C74 and C75 of tRNA^Glu^.

## Results

### Influence of tRNA on the interaction of GluRS with Glu-AMS

**Fig 2:**
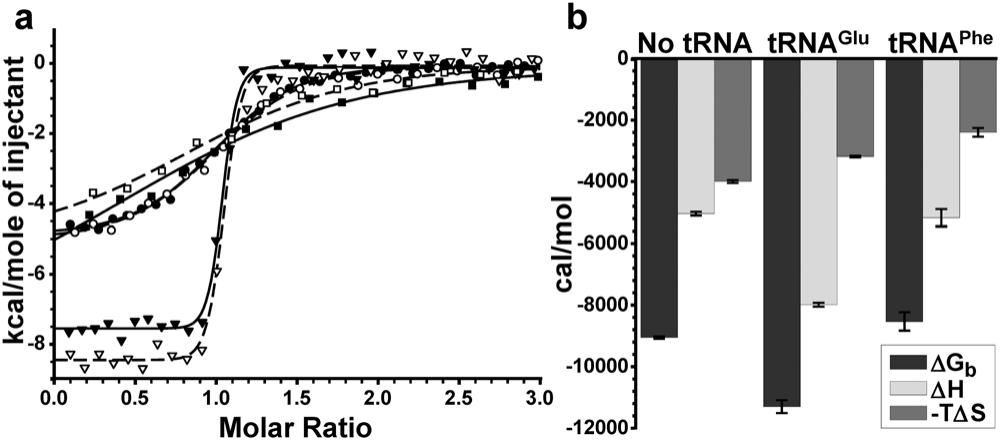
Glu-AMS binding to GluRS with/without tRNA. (a) Integrated ITC curves of Glu-AMS binding to GluRS with/without tRNA. Binding of Glu-AMS: to GluRS alone (circles), to GluRS with saturating concentration of tRNA^Glu^ in enriched total tRNA from *E*. *coli* (upside-down triangles), to GluRS with 11.23 µM tRNA^Phe^ from brewer’s yeast (squares). A duplicata of each tested condition is shown. The values shown in (b) were calculated from means of two distinct experiments reported in Table 1, weighting by inverse variance [32].

The thermodynamic parameters ΔG_b_, ΔH_b_ and -TΔS_b_ of the interaction of GluRS with Glu-AMS were measured at 30°C in the absence of tRNA and in the presence of a small excess of *E*. *coli* tRNA^Glu^ and, as a negative control, of the non-cognate tRNA^Phe^ from *S*. *cerevisiae* (Fig 2 and Table 1; all values and errors in this table were obtained by weighting by inverse variance [32], except for ΔG_b_ values and errors, obtained by simple average and standard error calculations).

A substantial entropic contribution for the interactions between Glu-AMS and GluRS in the absence of tRNA or in the presence of the cognate tRNA^Glu^ or of the non-cognate tRNA^Phe^ is indicated by the negative values of-TΔS_b_ under these three conditions (Fig 2). The importance of the entropic contribution is also revealed by the negative value of ΔC_p_ in the absence of tRNA (see below). On the other hand, the large negative enthalpy is the dominant contributor to the free energy of the Glu-AMS GluRS interaction in the absence of tRNA; it is not altered in the presence of the non-cognate tRNA^Phe^, but is strongly increased in the presence of the cognate tRNA^Glu^, resulting in an increase of the negative value of ΔG_b_ from -9050 to -11300 cal/mol) (Table 1). These results indicate that there are many favorable hydrogen bonds and/or van der Waals interactions between Glu-AMS and GluRS.

### Temperature-dependence of the GluRS/Glu-AMS interaction

**Fig 3:**
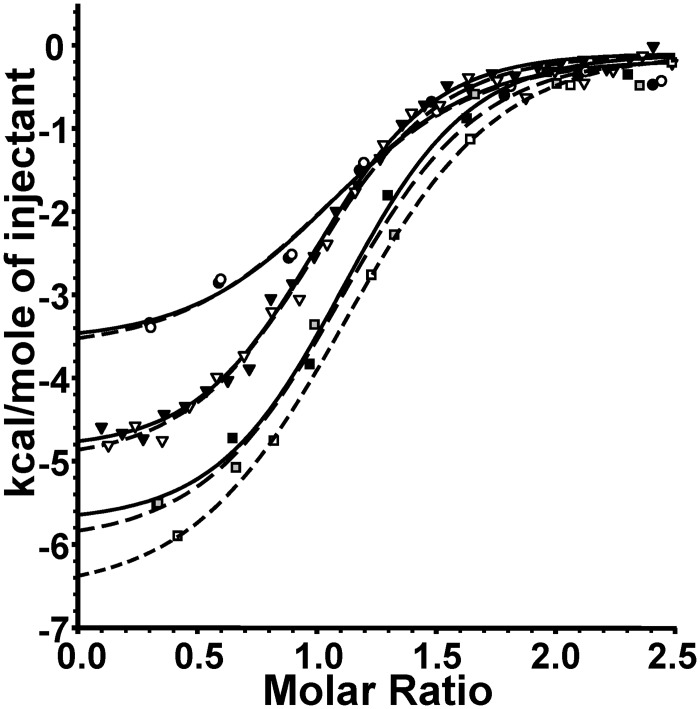
Temperature-dependence of Glu-AMS binding to GluRS. Integrated ITC curves of Glu-AMS (90 μM) binding to GluRS (9 μM) at different temperatures; 20°C (circles), 30°C (upside-down triangles), 37°C (squares).

The influence of temperature on Glu-AMS binding to GluRS was also investigated by microcalorimetry at 20, 30 and 37°C (*i*.*e*. 293, 303 and 310 K), in the absence of tRNA^Glu^ (Fig 3). The ΔG_b_ values are similar at these temperatures, but ΔH_b_ values increase with temperature (Table 2).

**Table 2:** Temperature-dependance of the GluRS Glu-AMS interaction.

T (K)	293	303	310
n	1.0097 ± 0.0369	0.9998 ± 0.0094	1.0016 ± 0.0095
ΔH_b_ (cal/mol)	-3929 ± 191	-5041 ± 63	-6395 ± 81
ΔS_b_ (cal/mol·K)	15.29 ± 0.74	13.19 ± 0.16	9.146 ± 0.117
ΔG_b_ (cal/mol)	-8396 ± 1.6	-9056 ± 38	-9326 ± 100
-TΔS_b_ (cal/mol)	-4479 ± 218	-3996 ± 50	-2835 ± 36

n = stoichiometry coefficient (number of moles of Glu-AMS bound per mole of GluRS monomer), ΔH_b_ = reaction enthalpy, ΔS_b_ = reaction entropy, T = temperature, ΔG_b_ = reaction energy (calculated with the formula ΔG_b_ = -RT Ln *K*_b_, where R = 1.987 cal/mol·K).

Injections of Glu-AMS at 90 μM were done in a starting concentration of 9 μM of GluRS.

Raw data and calculated values for each separated ITC runs are shown in S2 Table.

By plotting these ΔH_b_ values as a function of temperature (S2 Fig), we calculated the change in heat capacity (ΔC_p_) using the following equation: ΔC_p_ = (ΔH_T2_ - ΔH_T1_)/(T2—T1) [33]. The calculated value is -143 ± 23 cal/mol·K.

### Structural analysis of the GluRS Glu-AMS interaction

**Fig 4:**
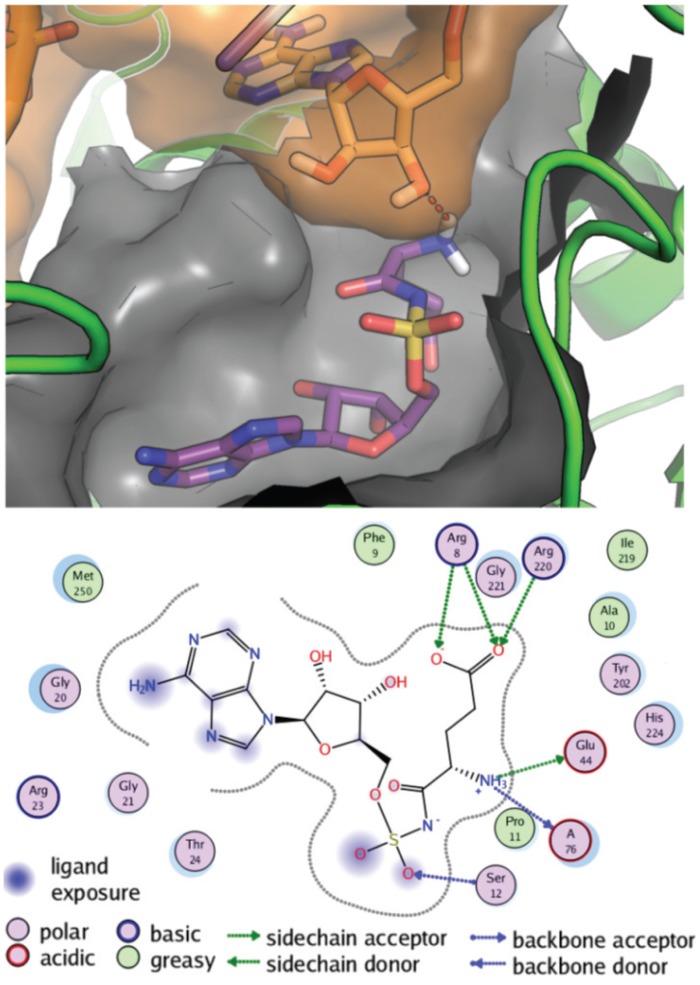
Molecular docking of Glu-AMS and *E*. *coli* tRNA^Glu^ on *E*. *coli* GluRS. Structural representation of the Glu-AMS and *E*. *coli* tRNA^Glu^ in the *E*. *coli* GluRS binding site from the docking simulations. Top: Glu-AMS is in purple sticks, *E*. *coli* tRNA^Glu^ A76 is in orange sticks and a transparent orange surface, *E*. *coli* GluRS is in green cartoon, and the binding site is depicted as a grey surface. The H-bond formed between Glu-AMS and A76 is shown as a dotted red line. Only the hydrogen atoms of the NH_3_ group involved in this H-bond are shown for clarity. Bottom: 2D representation of the Glu-AMS docked conformation bound to *E*. *coli* GluRS. The binding pocket is represented with a grey dotted line, polar and non-polar residues are represented as magenta and green circles, respectively, and polar interactions are shown as green and blue lines.

Homology models for *E*. *coli* GluRS, both in the absence and in the presence of *E*. *coli* tRNA^Glu^ were built from the *T*. *elongatus* GluRS crystal structure. Docking of Glu-AMS showed that the binding mode was conserved in both models. The RMSDs between the docked Glu-AMS conformations and the *T*. *thermophilus* crystal structures (PDB 2CV2) are 0.79 and 0.82 Å in the absence and in the presence of tRNA^Glu^, respectively. The docking result for *E*. *coli* GluRS•tRNA^Glu^ model is presented in Fig 4 for the *E*. *coli* GluRS•tRNA^Glu^ model, where the Glu-AMS NH_3_ moiety interacts with both GluRS Glu44 and with the 3’-OH group of tRNA^Glu^ A76, leaving free the vicinal 2’-OH group on which GluRS transfers the glutamyl group from Glu-AMP [34].

## Discussion

Aminoacyl adenylates (aa-AMP) are one of the products of the activation reaction catalyzed by aaRSs, and one of the substrates of the subsequent transfer reaction (reviewed by Giegé and Springer, 2012) [35]. Most aa-AMP are relatively stable when bound to their activating enzyme, and can be isolated in complex with a corresponding aaRS [36]. In 1963, Meister reported the synthesis of 16 aa-AMP, not including glutamyl-AMP [37]. The instability and transient existence of Glu-AMP in the formation of Glu-tRNA were revealed in kinetic studies of the reaction mechanism of *E*. *coli* GluRS [38]. Glu-AMS, an analogue of Glu-AMP, is the best known inhibitor of *E*. *coli* GluRS [6] probably because the dimensions of the sulfamoyl group are nearly the same as those of the phosphate group, and because it can exist in the anionic form, both in solution and in the solid state, due to the acidity of the NH function; the negative charge is delocalized over several atoms, and the anion is a good mimic of the phosphate ion [4].

The microcalorimetric study reported here reveals a substantial entropic contribution for the interactions between Glu-AMS and GluRS in the absence of tRNA or in the presence of the cognate tRNA^Glu^ or of a non-cognate tRNA^Phe^, indicated by the negative values of-TΔS_b_. The entropy term for Glu-AMS binding at 30°C (Table 1) to the free GluRS (the apo-enzyme) is greater (13.2 cal/mol·K) than for its binding to the GluRS/tRNA^Glu^ complex (the holoenzyme) (10.5 cal/mol·K). This result indicates that the active site is less crowded in the apoenzyme than in the holoenyme; the fit in the holoenzyme would have to be better than in the apoenzyme. In other words, the Glu-AMS would be more constrained in the holoenzyme, thus restricting motion. This would translate into a smaller entropy term. Such favorable entropic contribution is in agreement with the values reported for the binding of ATP, whose polarity is similar to that of Glu-AMS, to MEK1 [39] and to F_1_-ATPase [40]. A favorable entropic contribution was reported to arise from the desolvation of the polar binding site [41], as the polar residues forming the cavity constrain the water molecules in a stiff H-bond network. The negative value of ΔC_p_ during the binding of Glu-AMS to GluRS (S2 Fig) also suggests the breaking of a tight H-bond network, similarly to the hydrophobic interactions. Indeed, in the case of the hydrophobic effect, such a change in heat capacity of binding is thought to arise from the accommodation of non-polar groups by water [42,43].

On the other hand, the large negative enthalpy is the dominant contribution to the free energy of the Glu-AMS binding to GluRS in the absence of tRNA; it suggests that there are several favorable hydrogen bonds and/or van der Waals interactions between Glu-AMS and GluRS. The free energy is not altered in the presence of the non-cognate tRNA^Phe^, but is strongly increased in the presence of the cognate tRNA^Glu^. This increased binding of Glu-AMS to GluRS in the presence of the cognate tRNA^Glu^ results from the reorientation of the tRNA^Glu^ 3’ end, observed from PDB 2CV2 for the *T*. *thermophilus* GluRS [16], leading to an additional H-bond between the residue A76 and Glu-AMS (see S3 Fig), also confirmed for *E*. *coli* GluRS from docking simulations (Fig 4). Indeed, the free energy of binding difference of about 2.2 kcal/mol observed in the presence of tRNA^Glu^ (Table 1) is in the range of the known values for the strength of H-bonds [41]. The Glu-AMS•GluRS•tRNA^Glu^ complex is a posttransition-state mimic [16], *i*.*e*. a state following the glutamate activation reaction and preceding the attack by tRNA^Glu^ on Glu-AMP; in this posttransition-state, the stronger binding of Glu-AMS to GluRS in the presence than in the absence of tRNA^Glu^ revealed by our results, is due to a direct involvement of tRNA^Glu^ to the binding of Glu-AMS to the GluRS•tRNA^Glu^ complex.

Because of the high instability of the aminoacylation reaction intermediate Glu-AMP [44], it is very difficult to characterize its interaction with GluRS. The very high structural similarity between Glu-AMP and Glu-AMS allowed us to use the latter in structural studies which revealed that the presence of tRNA^Glu^ bound to GluRS is required for the correct positioning of the α-phosphate of ATP and of the α-COOH of glutamate for the catalysis of the activation reaction [16]. The 50-fold decrease in the affinity of GluRS for Glu-AMS in the absence of tRNA^Glu^ (Table 1 and Fig 2) suggests that the Glu-AMP GluRS interaction in the absence of tRNA^Glu^ is much weaker than that between other aaRSs and their cognate aa-AMP, and has the same order of magnitude as the interaction between a non-cognate aa-AMP and an aaRS, such as tyrosyl-AMP (Tyr-AMP) and phenylalanyl-tRNA synthetase (PheRS) (Table 3).[7,45–48] The released intermediate would likely be hydrolyzed very fast by one of the mechanisms of pre-transfer editing [49] (reviewed by Ling et al., 2009) [50]. This putative low affinity of GluRS for Glu-AMP could explain why this enzyme evolved to require the presence of its cognate tRNA to activate glutamate, allowing the immediate transfer of glutamate from Glu-AMP to the acceptor end of tRNA, and thus preventing unproductive cleavage of ATP. The fact that all the known GluRSs share this property [18] supports this model. The generality of this model could be tested by determining the influence of cognate and non-cognate tRNAs on the binding of each of the three other aaRSs, whose activation reaction is tRNA-dependent (GlnRS, ArgRS and class I LysRS), to the corresponding aminoacyl-sulfamoyl adenosine.

**Table 3:** Affinities of several aaRSs for cognate and non-cognate aa-AMP.

aaRS	aa-AMP or analogues	*K*_d_ aa-AMP aaRS	Reference
GluRS from *E*. *coli*	Glu-AMS	309 nM in the absence of tRNA^Glu^ 7 nM in the presence of tRNA^Glu^	This work^a^
PheRS from baker’s yeast	Phe-AMP	5 nM	[45]
	Tyr-AMP	1000 nM	[46]
ThrRS from yeast mitochodria	Threonyl-sulfamoyl-adenosine (Thr-AMS)	4.5 nM	[47]
	Seryl-sulfamoyl-adenosine (Ser-AMS)	450 nM	[47]
IleRS from *E*. *coli*	Ile-AMP and Val-AMP	This IleRS binds Val-AMP with a 150-fold weaker affinity than Ile-AMP.	[7]; reviewed by Fersht, 1998 [48]

^a^: These *K*_d_ values (dissociation constant) were calculated with the formula *K*_d_ = 1/*K*_b_.

## Supporting Information

S1 Fig Multiple sequence alignment of GluRS for *E*. *coli*, *B*. *thailandensis*, *T*. *elongatus* and *T*. *thermophilus*. (DOCX)

S2 Fig Graphical determination of ΔC_p_. (DOCX)

S3 Fig Structural comparison of *T*. *thermophilus* GluRS binding site without tRNA, with tRNA and with tRNA and Glu-AMS. (DOCX)

S1 Table Influence of tRNA on GluRS Glu-AMS binding at 30°C. (DOCX)

S2 Table Temperature-dependence of the GluRS Glu-AMS interaction. (DOCX)
