# Correction: tRNAGlu Increases the Affinity of Glutamyl-tRNA Synthetase for Its Inhibitor Glutamyl-Sulfamoyl-Adenosine, an Analogue of the Aminoacylation Reaction Intermediate Glutamyl-AMP: Mechanistic and Evolutionary Implications

**DOI:** 10.1371/journal.pone.0130238

**Published:** 2015-06-03

**Authors:** Sébastien P. Blais, Jack A. Kornblatt, Xavier Barbeau, Guillaume Bonnaure, Patrick Lagüe, Robert Chênevert, Jacques Lapointe

There is an error in the published article. The article should read as follows:

The following nomenclature was used to describe the interaction at equilibrium between Glu-AMS and either GluRS or a GluRS•tRNA complex:

GluRS•Glu-AMS↔GluRS+Glu-AMS

$$K_{\text{d}}=\left[\text{GluRS}\right]\times \left[\text{Glu-AMS}\right]/\left[\text{GluRS}•\text{Glu-AMS}\right]$$

$$1/K_{\text{d}}=K_{\text{b}}\;\left(\text{binding constant},\;\text{sometimes referred to as}\;K_{\text{a}}\right).$$
