# Supplementary material for: tRNAGlu Increases the Affinity of Glutamyl-tRNA Synthetase for Its Inhibitor Glutamyl-Sulfamoyl-Adenosine, an Analogue of the Aminoacylation Reaction Intermediate Glutamyl-AMP: Mechanistic and Evolutionary Implications
Source: PLoS One. 2015 Apr 10;10(4):e0121043. doi: 10.1371/journal.pone.0121043 (PMC4393105; doi:10.1371/journal.pone.0121043)
Supplement: S1 Fig — (DOCX) [file pone.0121043.s001.docx]

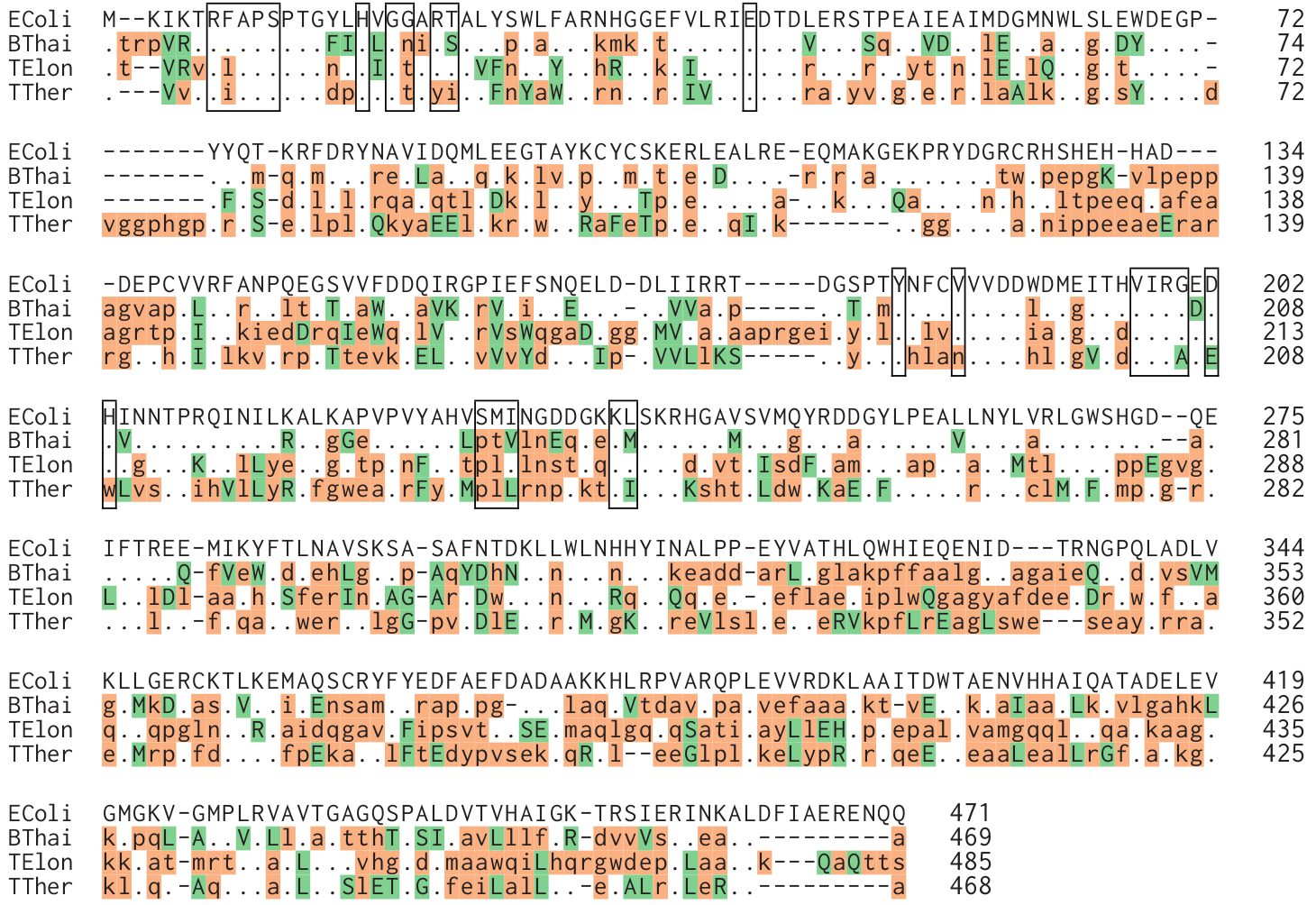


**S1 Figure:** Multiple sequence alignment of GluRS for *E. coli*, *B. thailandensis*, *T. elongatus* and *T. thermophilus* (Uniprot: P04805, Q2SX36, Q8DLI5 and P27000). Identical residues are represented as dots ("."), similar residues are colored in green and diverging residues in orange. Residues within 5 Å of Glu-AMS in 2CV2 are highlighted with a black box. Numbering for each sequence is on the right.
