# Supplementary material for: tRNAGlu Increases the Affinity of Glutamyl-tRNA Synthetase for Its Inhibitor Glutamyl-Sulfamoyl-Adenosine, an Analogue of the Aminoacylation Reaction Intermediate Glutamyl-AMP: Mechanistic and Evolutionary Implications
Source: PLoS One. 2015 Apr 10;10(4):e0121043. doi: 10.1371/journal.pone.0121043 (PMC4393105; doi:10.1371/journal.pone.0121043)
Supplement: S2 Fig — (DOCX) [file pone.0121043.s002.docx]

**

**

**S2 Figure:** Graphical determination of ΔC_p_. The ΔH_b_ values for the Glu-AMS GluRS interaction were taken from Figure 3, and plotted as a function of temperature. ΔC_p_ = (ΔH_T2_ - ΔH_T1_)/(T2 - T1) = -143 ± 23 cal/mol·K.
