# Supplementary material for: tRNAGlu Increases the Affinity of Glutamyl-tRNA Synthetase for Its Inhibitor Glutamyl-Sulfamoyl-Adenosine, an Analogue of the Aminoacylation Reaction Intermediate Glutamyl-AMP: Mechanistic and Evolutionary Implications
Source: PLoS One. 2015 Apr 10;10(4):e0121043. doi: 10.1371/journal.pone.0121043 (PMC4393105; doi:10.1371/journal.pone.0121043)
Supplement: S3 Fig — (DOCX) [file pone.0121043.s003.docx]

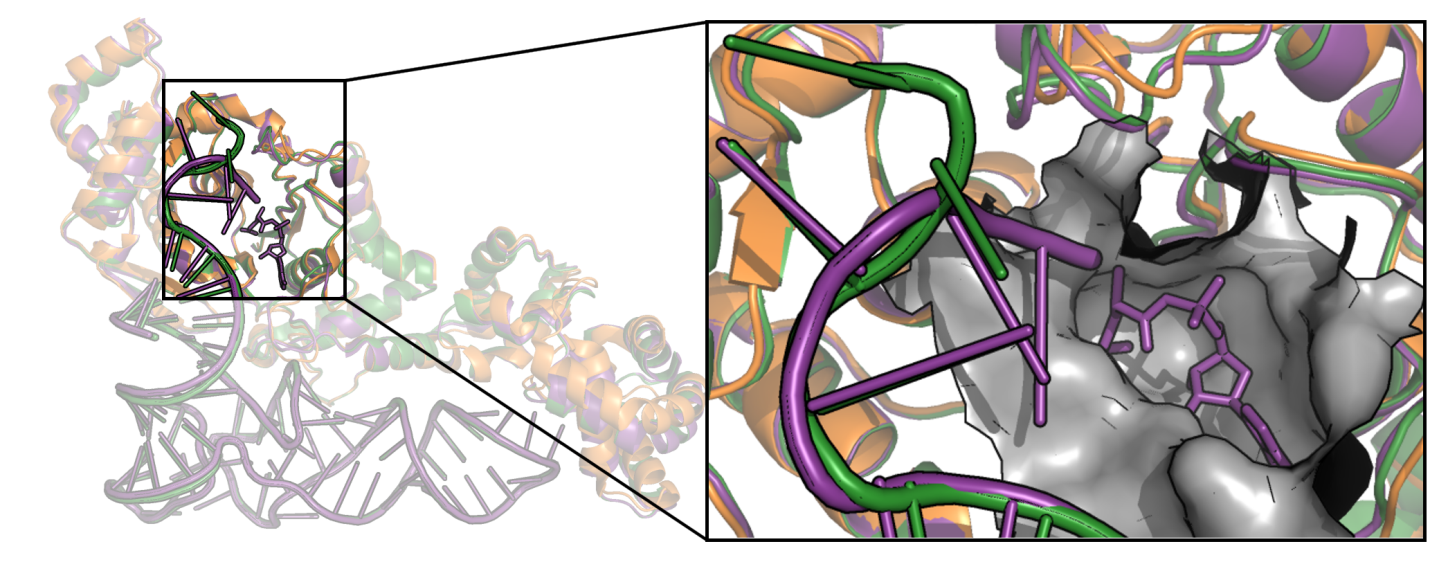


**S3 Figure:** Structural comparison of *T. thermophilus* GluRS binding site without tRNA (PDB 2CUZ, orange), with tRNA (PDB 1G59, green) and with tRNA and Glu-AMS (PDB 2CV2, purple). GluRS is shown in cartoon, Glu-AMS as purple sticks and the residues from the active site are in grey surface.
