# Supplementary material for: tRNAGlu Increases the Affinity of Glutamyl-tRNA Synthetase for Its Inhibitor Glutamyl-Sulfamoyl-Adenosine, an Analogue of the Aminoacylation Reaction Intermediate Glutamyl-AMP: Mechanistic and Evolutionary Implications
Source: PLoS One. 2015 Apr 10;10(4):e0121043. doi: 10.1371/journal.pone.0121043 (PMC4393105; doi:10.1371/journal.pone.0121043)
Supplement: S1 Table — (DOCX) [file pone.0121043.s004.docx]

**S1 Table:** Influence of tRNA on GluRS Glu-AMS binding at 30°C. Raw data and calculated values for each separate ITC runs.

| [ligand] (µM) | 90 | 90 | 90 | 45 | 90 | 90 |
| --- | --- | --- | --- | --- | --- | --- |
| [GluRS] (µM) | 5.355 | 4.143 | 5.792 | 2.636 | 2.434 | 2.198 |
| [tRNA] (µM) | n.a. | n.a. | tRNA^Glu^ 10.6 | tRNA^Glu^ 5.3 | tRNA^Phe^ 11.23 | tRNA^Phe^ 11.23 |
| n^a^ | 1.001 ± 0.011 | 0.9963 ± 0.0184 | 1.000 ± 0.006 | 1.004 ± 0.006 | 0.9835 ± 0.0809 | 0.9925 ± 0.048 |
| *K*_b_ (M^-1^) | 3.258×10^6^ ± 2.926×10^5^ | 3.563×10^6^ ± 4.962×10^5^ | 1.116×10^8^ ± 2.817×10^7^ | 1.822×10^8^ ± 4.478×10^7^ | 1.013×10^6^ ± 1.950×10^5^ | 2.092×10^6^ ± 3.746×10^5^ |
| ΔH_b_ (cal/mol) | -5007 ± 73 | -5142 ± 127 | -7610 ± 81 | -8537 ± 97 | -6657 ± 689 | -4882 ± 305 |
| ΔS_b_ (cal/mol·K) | 13.26 ± 0.19 | 12.99 ± 0.32 | 11.71 ± 0.12 | 9.596 ± 0.11 | 5.472 ± 0.57 | 12.79 ± 0.80 |
| Temperature (K) | 303 | 303 | 303 | 303 | 303 | 303 |
| *K*_d_ (nM) | 307 ± 28 | 281 ± 39 | 8.96 ± 2.26 | 5.49 ± 1.35 | 987 ± 190 | 478 ± 86 |
| ΔG_b_ (cal/mol) | -9029 | -9083 | -11156 | -11452 | -8326 | -8762 |
| -TΔS_b_ | -4018 ± 58 | -3936 ± 97 | -3548 ± 38 | -2908 ± 33 | -1658 ± 172 | -3875 ± 242 |

Ligand = Glutamyl-sulfamoyl-adenosine (Glu-AMS), n = stoichiometry coefficient (number of moles of Glu-AMS bound per mole of GluRS monomer), *K*_b_ = binding constant, ΔH_b_ = reaction enthalpy, ΔS = reaction entropy, *K*_d_ = dissociation constant (calculated with the formula *K*_d_ = 1/*K*_b_), ΔG_b_ = reaction energy (calculated with the formula ΔG_b_ = -RT Ln *K*_b_, where R (ideal gas constant) = 1.987 cal/mol·K).

Errors for n, *K*_b_ and ΔH are given by the Origin Software. Error for ΔS_b_ is the same relative error as for ΔH_b_, this is also the error carried out in –TΔS_b_ calculations. Error for *K*_d_ is the same relative error as for *K*_b_.

^a^ In the first analysis, n was fluctuating in the range of 0.27 to 0.72. Several factors may contribute to the fact that n, the number of mole of Glu-AMS bound per mole of GluRS, is smaller than the expected value of 1. First, the spectrophotometric determination of the concentration of this enzyme overestimates by about 10% the concentration of active sites, as revealed by active site titration.[[1](#_ENREF_1)] Secondly, several GluRS conformers have been observed in the crystal structures of *T. thermophilus* GluRS and of the GluRS•tRNA^Glu^ complex;[[2](#_ENREF_2)] the different values of n in these experimental conditions, the absence of tRNA or in the presence of tRNA^Glu^ or of tRNA^Phe^, may be due to the presence of GluRS conformers inactive for binding Glu-AMS, and whose proportion would differ under these three conditions. To correct this we took the analysis as it has been carried out in the first place and in which n is significantly less than 1. We multiplied the GluRS concentration by the n value and entered this new value as the macromolecule concentration in the analysis software. If the concentration entered were, say 0.008 mM, 0.63 × 0.008 mM would give the new concentration, 0.00504 mM. We repeated the complete fitting to one site making sure that n, *K*_b_ and ΔH were allowed to float. The new value of n is now close to 1.

1. Kern D, Lapointe J (1980) The catalytic mechanism of glutamyl-tRNA synthetase of *Escherichia coli*. Evidence for a two-step aminoacylation pathway, and study of the reactivity of the intermediate complex. European Journal of Biochemistry 106: 137-150.

2. Sekine S-i, Nureki O, Dubois DY, Bernier S, Chênevert R, Lapointe J, et al. (2003) ATP binding by glutamyl-tRNA synthetase is switched to the productive mode by tRNA binding. EMBO Journal 22: 676-688.
