# Supplementary material for: tRNAGlu Increases the Affinity of Glutamyl-tRNA Synthetase for Its Inhibitor Glutamyl-Sulfamoyl-Adenosine, an Analogue of the Aminoacylation Reaction Intermediate Glutamyl-AMP: Mechanistic and Evolutionary Implications
Source: PLoS One. 2015 Apr 10;10(4):e0121043. doi: 10.1371/journal.pone.0121043 (PMC4393105; doi:10.1371/journal.pone.0121043)
Supplement: S2 Table — (DOCX) [file pone.0121043.s005.docx]

**S2 Table:** Temperature-dependance of the GluRS Glu-AMS interaction. Raw data and calculated values for each separate ITC runs.

| [ligand] (µM) | 90 | 90 | 90 | 90 | 90 | 90 | 90 |
| --- | --- | --- | --- | --- | --- | --- | --- |
| [GluRS] (µM) | 4.15 | 4.35 | 5.355 | 4.143 | 4.015 | 3.931 | 3.169 |
| n^a^ | 1.014 ± 0.054 | 1.006 ± 0.050 | 1.001 ± 0.011 | 0.9963 ± 0.0184 | 1.006 ± 0.019 | 1.005 ± 0.037 | 0.9997 ± 0.0114 |
| *K*_b_ (M^-1^) | 1.836×10^6^ ± 5.461×10^5^ | 1.829×10^6^ ± 5.002×10^5^ | 3.258×10^6^ ± 2.926×10^5^ | 3.563×10^6^ ± 4.962×10^5^ | 4.335×10^6^ ± 6.890×10^5^ | 3.149×10^6^ ± 8.103×10^5^ | 3.904×10^6^ ± 3.109×10^5^ |
| ΔH_b_ (cal/mol) | -3909 ± 279 | -3946 ± 263 | -5007 ± 73 | -5142 ± 127 | -5873 ± 150 | -6225 ± 307 | -6654 ± 102 |
| ΔS_b_ (cal/mol·K) | 15.34 ± 1.09 | 15.24 ± 1.01 | 13.26 ± 0.19 | 12.99 ± 0.32 | 11.39 ± 0.29 | 9.607 ± 0.473 | 8.646 ± 0.132 |
| Temperature (K) | 293 | 293 | 303 | 303 | 310 | 310 | 310 |
| *K*_d_ (nM) | 545 ± 162 | 547 ± 150 | 307 ± 28 | 281 ± 39 | 231 ± 37 | 318 ± 82 | 256 ± 20 |
| ΔG_b_ (cal/mol) | -8397 | -8395 | -9029 | -9083 | -9413 | -9217 | -9349 |
| -TΔS_b_ | -4495 ± 320 | -4465 ± 297 | -4018 ± 58 | -3936 ± 97 | -3531 ± 90 | -2978 ± 147 | -2680 ± 41 |

Ligand = Glutamyl-sulfamoyl-adenosine (Glu-AMS), n = stoichiometry coefficient (number of moles of Glu-AMS bound per mole of GluRS monomer), *K*_b_ = binding constant, ΔH_b_ = reaction enthalpy, ΔS = reaction entropy, *K*_d_ = dissociation constant (calculated with the formula *K*_d_ = 1/*K*_b_), ΔG_b_ = reaction energy (calculated with the formula ΔG_b_ = -RT Ln *K*_b_, where R (ideal gas constant) = 1.987 cal/mol·K).

Errors for n, *K*_b_ and ΔH are given by the Origin Software. Error for ΔS_b_ is the same relative error as for ΔH_b_, this is also the error carried out in –TΔS_b_ calculations. Error for *K*_d_ is the same relative error as for *K*_b_.

^a^ In the first analysis, n was fluctuating in the range of 0.27 to 0.72. Several factors may contribute to the fact that n, the number of mole of Glu-AMS bound per mole of GluRS, is smaller than the expected value of 1. First, the spectrophotometric determination of the concentration of this enzyme overestimates by about 10% the concentration of active sites, as revealed by active site titration.[[1](#_ENREF_1)] Secondly, several GluRS conformers have been observed in the crystal structures of *T. thermophilus* GluRS and of the GluRS•tRNA^Glu^ complex;[[2](#_ENREF_2)] the different values of n in these experimental conditions, the absence of tRNA or in the presence of tRNA^Glu^ or of tRNA^Phe^, may be due to the presence of GluRS conformers inactive for binding Glu-AMS, and whose proportion would differ under these three conditions. To correct this we took the analysis as it has been carried out in the first place and in which n is significantly less than 1. We multiplied the GluRS concentration by the n value and entered this new value as the macromolecule concentration in the analysis software. If the concentration entered were, say 0.008 mM, 0.63 × 0.008 mM would give the new concentration, 0.00504 mM. We repeated the complete fitting to one site making sure that n, *K*_b_ and ΔH were allowed to float. The new value of n is now close to 1.

1. Kern D, Lapointe J (1980) The catalytic mechanism of glutamyl-tRNA synthetase of *Escherichia coli*. Evidence for a two-step aminoacylation pathway, and study of the reactivity of the intermediate complex. European Journal of Biochemistry 106: 137-150.

2. Sekine S-i, Nureki O, Dubois DY, Bernier S, Chênevert R, Lapointe J, et al. (2003) ATP binding by glutamyl-tRNA synthetase is switched to the productive mode by tRNA binding. EMBO Journal 22: 676-688.
